# The impacts of maternal mortality and cause of death on children’s risk of dying in rural South Africa: evidence from a population based surveillance study (1992-2013)

**DOI:** 10.1186/1742-4755-12-S1-S7

**Published:** 2015-05-06

**Authors:** Brian Houle, Samuel J Clark, Kathleen Kahn, Stephen Tollman, Alicia Ely Yamin

**Affiliations:** 1 Australian Demographic and Social Research Institute, The Australian National University, Canberra, Australia; 2 Institute of Behavioral Science, University of Colorado at Boulder, Boulder, Colorado, USA; 3 MRC/Wits Rural Public Health and Health Transitions Research Unit (Agincourt), School of Public Health, Faculty of Health Sciences, University of the Witwatersrand, Johannesburg, South Africa; 4 Department of Sociology, University of Washington, Seattle, Washington, USA; 5 INDEPTH Network, Accra, Ghana; 6 Centre for Global Health Research, Umeå University, Umeå, Sweden; 7 François-Xavier Bagnoud Center for Health and Human Rights, Harvard School of Public Health, Boston, MA, USA; 8 Department of Global Health and Population, Harvard School of Public Health, Boston, MA, USA

**Keywords:** Maternal mortality; HIV; Child mortality; Infant mortality; South Africa; rural; health and demographic surveillance system

## Abstract

**Background:**

Maternal mortality, the HIV/AIDS pandemic, and child survival are closely linked. This study contributes evidence on the impact of maternal death on children’s risk of dying in an HIV-endemic population in rural South Africa.

**Methods:**

We used data for children younger than 10 years from the Agincourt health and socio-demographic surveillance system (1992 – 2013). We used discrete time event history analysis to estimate children’s risk of dying when they experienced a maternal death compared to children whose mother survived (N=3,740,992 child months). We also examined variation in risk due to cause of maternal death. We defined mother’s survival status as early maternal death (during pregnancy, childbirth, or within 42 days of most recent childbirth or identified cause of death), late maternal death (within 43-365 days of most recent childbirth), any other death, and mothers who survived.

**Results:**

Children who experienced an early maternal death were at 15 times the risk of dying (RRR 15.2; 95% CI 8.3–27.9) compared to children whose mother survived. Children under 1 month whose mother died an early (p=0.002) maternal death were at increased risk of dying compared to older children. Children whose mothers died of an HIV/AIDS or TB-related early maternal death were at 29 times the risk of dying compared to children with surviving mothers (RRR 29.2; 95% CI 11.7–73.1). The risk of these children dying was significantly higher than those children whose mother died of a HIV/AIDS or TB-related non-maternal death (p=0.017).

**Conclusions:**

This study contributes further evidence on the impact of a mother’s death on child survival in a poor, rural setting with high HIV prevalence. The intersecting epidemics of maternal mortality and HIV/AIDS – especially in sub-Saharan Africa – have profound implications for maternal and child health and well-being. Such evidence can help guide public and primary health care practice and interventions.

## Background

To reduce child mortality and improve maternal health (including reducing maternal mortality and increasing access to reproductive health) are central Millennium Development Goals. The maternal mortality ratio (MMR) increased rapidly in sub-Saharan Africa since 1990 and only recently has begun to decline [1]. Estimates from the World Health Organization and partners suggests the region accounted for 62% of global maternal deaths in 2013 [2]. Levels of child mortality in sub-Saharan Africa remain the highest in the world – comprising almost half of global under-five deaths [3]. This is compounded by projections that the child population is expected to increase rapidly in the next 20 years [3].

Prior research has begun to explore the impacts of maternal death on child outcomes [4-8]. However, additional evidence is needed. Study results may be limited in terms of cross-cultural generalizability, particularly given differences in household organization, available resources, and care taking practices. This study seeks to add to this emerging literature by examining the effect of a maternal death on child survival in rural South Africa. While an earlier analysis examined the relationship between the timing of a mother’s death and risk of child mortality – it did not isolate the risk due to maternal mortality [8]. This study further extends these results by including older children (up to age 10 years versus 5 years) and cause of child deaths.

Further research is also needed on the connection between maternal and child mortality and the HIV pandemic. While the biological pathways relating HIV and maternal mortality are not fully understood [9,10], evidence suggests HIV is associated with global maternal mortality [11,12]. Maternal mortality and HIV are further linked to child health and wellbeing [13,14], for both HIV-infected and HIV-negative children of HIV-positive mothers [15]. This study contributes evidence on this relationship and children’s risk of dying in a setting with high prevalence of HIV/AIDS [16].

Our primary aim is to investigate the relationship between a child’s risk of dying and maternal death in rural South Africa. We leverage an extensive and robust data set – derived from the Agincourt health and demographic surveillance system – which covers the Agincourt sub-district in northeast South Africa and has conducted annual household surveys for 22 years. We investigate how a maternal death, using different definitions to capture immediate and longer-term effects, changes the risk of dying compared to children with surviving mothers and controlling for a variety of covariates. We also examine if these relationships vary by the mother’s cause of death, comparing deaths related to HIV/AIDS or tuberculosis (TB) to other causes. Finally, we explore the causes of child death according to their mother’s survival status.

## Methods

### Data and study population

We used data from the Agincourt health and demographic surveillance system (HDSS) from 1992 – 2013. The HDSS describes the population living in the Bushbuckridge subdistrict of Ehlanzeni district, Mpumalanga Province in northeast South Africa. Infrastructure in the area is limited, and unemployment is high. Almost a quarter of women ages 15+ and 4.4% of children 1–4 years are HIV-positive [16,17]. Mortality has increased in children and young and middle-aged adults [18,19,8]. Within the sub-district there are eight primary care clinics with the nearest district hospital 25 kilometers away. Data are available from the INDEPTH network iShare repository (http://www.indepth-ishare.org/index.php/home) as well as sample data from the Agincourt HDSS (http://www.agincourt.co.za/).

In 2011 the surveillance population was approximately 90,000 people residing in 27 rural villages. Every year trained fieldworkers interviewed the most knowledgeable person in each household and collected information on vital events, migration, nuptial events, and other information [20,21]. A specially trained team administered a validated verbal autopsy (VA) interview for each death in the household [22]. Every other year since 2001 fieldworkers also collected information on household socio-economic status (SES) using a validated, 34-item asset survey [23] – including information on living conditions (e.g., water access, electricity) and household assets (e.g., ownership of appliances and modern assets).

We included data for all children up to 120 months of age who were born from 1992 – 2013 (excluding stillbirths). To assess the effect of maternal death on child survival, we included information on the mother’s survival status, identifying: early maternal death (during pregnancy, childbirth, or within 42 days of most recent childbirth or identified cause of death), late maternal death (within 43-365 days of most recent childbirth), any other death, and mothers who survived. We operationalized household SES by taking quintiles of the absolute SES asset scale derived from household assets as in [19]. We included cause of death information for both the children and their mothers by analyzing the VA interviews using InterVA-4 [24,25]. InterVA-4 is an automated algorithm for assigning cause of death from VA data. Binary-coded VA data describing signs/symptoms leading up to death are combined with independently-generated data from physicians describing the relationship between signs/symptoms and causes to yield a numerical propensity of dying from each cause. These propensities are used to identify the two or three most ‘likely’ causes of death when at least one cause’s propensity is much larger than all the rest. This approach has the key advantage of being consistent and reproducible, addressing the fact that VA causes coded by physicians, the traditional alternative to automated approaches, include a physician-specific bias and are not generally replicable [26]. InterVA has been used extensively to interpret VA data [27] including at the Agincourt HDSS [28,19]. We specified the model with high HIV prevalence and low malaria prevalence. We used this approach instead of a physician-based VA approach to ensure consistent coding of causes of death and comparability across time.

### Statistical analysis

We analyzed child mortality using discrete time event history analysis [29,30] – where a child was at risk of dying for each month they were observed (up to and including death or censoring). We set covariate values at the beginning of each child month. We modeled the monthly probability of a child dying using relative risk regression [31], accounting for intra-mother correlation and including time constant covariates of child sex and mother’s age at birth; and time varying covariates of child age, year, and mother’s survival status. For instance, if a child’s mother died when the child turned 15 months old, their mother’s survival status would be coded as ‘survives’ for child months 0-14 and then coded as ‘other death’ from months 15+ where the child was observed. We tested if interactions of child sex or age and mother’s survival status improved overall model fit using the Bayesian Information Criterion (BIC) [32]. To assess if the effect of the type of death varied by cause, we also included a model with mother cause of death, categorized as HIV/AIDS or TB and all other causes. We also fit a model including household SES to determine if variation in SES explained the effect of mother’s survival status. We tested this association separately since household SES was only available from 2001, shortening the timespan of the data and reducing the sample size (for a detailed examination of household SES related to child mortality at this HDSS, see [14]). We compared causes of child death by the mother’s survival status using Fisher’s exact test, categorizing deaths according to Ronsmans et al. [4]. We completed all analyses using Stata [33].

### Ethical approval

The Agincourt health and socio-demographic surveillance system (HDSS) was reviewed and approved by the University of the Witwatersrand Human Research Ethics Committee (Medical) (protocol M110138 (previously M960720) and M081145). Informed consent is obtained for individuals and households at each follow-up visit.

## Results

Table 1 shows characteristics of children included in this study. From 1992 – 2013 there were 70,418 live births and 1,747 deaths to children under 10 years of age. The median age of mothers at birth was 24 years. Eighty-four children had a mother who died due to an early maternal death and 100 had a mother who died to a late maternal death. Seventy-one mothers died due to an early maternal death, 87 to a late maternal death, and 1,376 to other causes.

**Table 1:** Characteristics of children according to mother’s survival status, Agincourt health and demographic surveillance system, South Africa, 1992 – 2013. Data reported as number (%). Early maternal death defined as death during pregnancy, childbirth, or within 42 days of most recent childbirth or identified cause of death, and late maternal death as death from 43 to 365 days after childbirth. Asset quintiles reported from last child observation (measurement began in 2001). Number of mothers dying due to early maternal, late maternal, other death, or who survived were 71, 87, 1376, and 35,752, respectively.

	Survives (n= 69, 637)	Early maternal death (n= 84)	Late maternal death (n= 100)	Other death (n= 597)	All (n= 70,418)
Sex					
Female	34827 (50)	39 (46)	47 (47)	304 (51)	35217 (50)
Male	34810 (50)	45 (54)	53 (53)	293 (49)	35201 (50)
Vital status					
Dies	1703 (2)	14 (17)	13 (13)	17 (3)	1747 (2)
Survives	67934 (98)	70 (83)	87 (87)	580 (97)	68671 (98)
Mother’s age (years)					
15 – 19	16584 (24)	17 (20)	14 (14)	84 (14)	16699 (24)
20 – 24	19853 (29)	18 (21)	23 (23)	154 (26)	20048 (28)
25 – 29	14714 (21)	23 (27)	30 (30)	142 (24)	14909 (21)
30 – 34	9816 (14)	16 (19)	15 (15)	109 (18)	9956 (14)
35 – 39	5728 (8)	7 (8)	8 (8)	69 (12)	5812 (8)
≥40	2942 (4)	3 (4)	10 (10)	39 (7)	2994 (4)
Asset quintiles					
Poorest	5590 (20)	13 (37)	8 (21)	96 (24)	5707 (21)
Less poor	5203 (19)	6 (17)	8 (21)	78 (20)	5295 (19)
Middle	5259 (19)	6 (17)	7 (18)	85 (22)	5357 (19)
Richer	5580 (20)	6 (17)	7 (18)	71 (18)	5664 (20)
Richest	5684 (21)	4 (11)	9 (23)	63 (16)	5760 (21)

The results from the relative risk regression estimations are shown in Table 2. An interaction between child sex and mother’s survival status did not improve model fit according to the BIC. An interaction between child age and mother’s survival status resulted in small cell sizes and was not included. The relative risk ratios describe the changes in a child’s risk of dying as a function of child sex, age, year, mother’s age at birth, and mother’s survival status and cause of death (Table 2). Male children had an increased risk of dying compared to females. Neonates and children under one year of age were at increased risk of dying relative to children 1–4 years old; while children ages 5–9 years were at decreased risk of dying. The risk of dying increased from 1999–2011 when HIV became highly prevalent, relative to 1992. For instance, in 2000 the risk of dying increased 74% compared to 1992. Since 2011 mortality risk has declined to pre-1999 levels.

**Table 2:** Relative risk regression of child death on child and mother characteristics, Agincourt health and demographic surveillance system, South Africa, 1992 – 2013 (n=3,740,992 child months). Adjusted for clustering of children with the same mothers. Early maternal death defined as death during pregnancy, childbirth, or within 42 days of most recent childbirth or identified cause of death, and late maternal death as death from 43 to 365 days after childbirth. Mother cause of death classified by InterVA-4 based on VA interviews. Model B includes an interaction of mother’s survival status with cause of death.

	Relative risk ratio	Model A 95% CI	p-value	Relative risk ratio	Model B 95% CI	p-value
Male	1.119	[1.016, 1.233]	0.023	1.118	[1.015, 1.232]	0.024
Child age (months)						
0	20.560	[17.858, 23.672]	<0.001	20.578	[17.873, 23.692]	<0.001
1-5	5.127	[4.508, 5.832]	<0.001	5.137	[4.516, 5.843]	<0.001
6-11	3.165	[2.746, 3.648]	<0.001	3.167	[2.748, 3.651]	<0.001
12-59	1.000	[1.000, 1.000]	.	1.000	[1.000, 1.000]	.
60-119	0.163	[0.134,0.198]	<0.001	0.163	[0.134, 0.198]	<0.001
Mother’s age (years)						
15-19	1.000	[1.000, 1.000]	.	1.000	[1.000, 1.000]	.
20-24	1.065	[0.930, 1.219]	0.366	1.062	[0.927, 1.217]	0.384
25-29	1.155	[1.000, 1.333]	0.050	1.153	[0.998, 1.331]	0.053
30-34	1.004	[0.852, 1.182]	0.966	1.007	[0.855, 1.186]	0.935
35-39	1.071	[0.888, 1.293]	0.473	1.075	[0.891, 1.297]	0.451
≥40	0.872	[0.672, 1.132]	0.303	0.870	[0.670, 1.130]	0.296
Year						
1992	1.000	[1.000, 1.000]	.	1.000	[1.000, 1.000]	.
1993	1.270	[0.755, 2.138]	0.368	1.272	[0.756, 2.140]	0.365
1994	1.147	[0.673, 1.956]	0.614	1.152	[0.676, 1.964]	0.603
1995	1.151	[0.677, 1.956]	0.603	1.158	[0.681, 1.968]	0.588
1996	0.971	[0.563, 1.676]	0.916	0.976	[0.566, 1.685]	0.932
1997	1.028	[0.599, 1.764]	0.921	1.031	[0.601, 1.769]	0.913
1998	1.543	[0.920, 2.587]	0.100	1.548	[0.923, 2.597]	0.098
1999	1.687	[1.016, 2.803]	0.043	1.697	[1.021, 2.821]	0.041
2000	1.740	[1.047, 2.891]	0.033	1.748	[1.052, 2.906]	0.031
2001	1.765	[1.060, 2.938]	0.029	1.774	[1.065, 2.954]	0.028
2002	2.456	[1.492, 4.044]	<0.001	2.468	[1.499, 4.065]	<0.001
2003	2.732	[1.668, 4.475]	<0.001	2.733	[1.669, 4.474]	<0.001
2004	1.876	[1.129, 3.118]	0.015	1.888	[1.136, 3.138]	0.014
2005	1.914	[1.159, 3.162]	0.011	1.926	[1.166, 3.183]	0.011
2006	2.112	[1.283, 3.477]	0.003	2.110	[1.282, 3.475]	0.003
2007	2.563	[1.567, 4.191]	<0.001	2.567	[1.570, 4.198]	<0.001
2008	2.924	[1.796, 4.759]	<0.001	2.938	[1.805, 4.783]	<0.001
2009	1.878	[1.139, 3.099]	0.014	1.897	[1.150, 3.131]	0.012
2010	1.687	[1.019, 2.792]	0.042	1.704	[1.029, 2.822]	0.038
2011	1.583	[0.953, 2.630]	0.076	1.598	[0.962, 2.656]	0.070
2012	1.062	[0.620, 1.819]	0.826	1.071	[0.625, 1.835]	0.803
2013	1.052	[0.537, 2.060]	0.882	1.058	[0.540, 2.072]	0.869
Mother’s survival status						
Survives	1.000	[1.000, 1.000]	.			
Early maternal death	15.169	[8.248, 27.898]	<0.001			
Late maternal death	13.567	[7.537, 24.421]	<0.001			
Other death	8.451	[5.138, 13.899]	<0.001			
Mother’s survival status × mother’s cause of death						
Survives				1.000	[1.000, 1.000]	.
HIV/AIDS or TB early maternal death				29.220	[11.688, 73.049]	<0.001
HIV/AIDS or TB late maternal death				14.332	[6.924, 29.665]	<0.001
HIV/AIDS or TB other death				7.477	[3.931, 14.222]	<0.001
Non-HIV/AIDS or TB early maternal death				9.197	[3.908, 21.647]	<0.001
Non-HIV/AIDS or TB late maternal death				12.453	[4.743, 32.697]	<0.001
Non-HIV/AIDS or TB other death				10.358	[4.772, 22.483]	<0.001

### Maternal death

The relative risk ratios corresponding to mother’s survival status in Table 2 (Model A) describe how a child’s risk of dying changes according to the survival status of their mother, adjusted for child sex and age, year, and mother’s age. Children who had a mother die an early maternal death were at 15 times the risk of dying (RRR 15.2; 95% CI 8.3–27.9), and those whose mother died a late maternal death were at 14 times the risk of dying (RRR 13.6; 95% CI 7.5–24.4), relative to children whose mother survived. Children who had a mother die any other type of death were at approximately 8 times the risk of dying (RRR 8.5 95% CI 5.1–13.9) relative to children whose mother survived.

**Figure 1:**
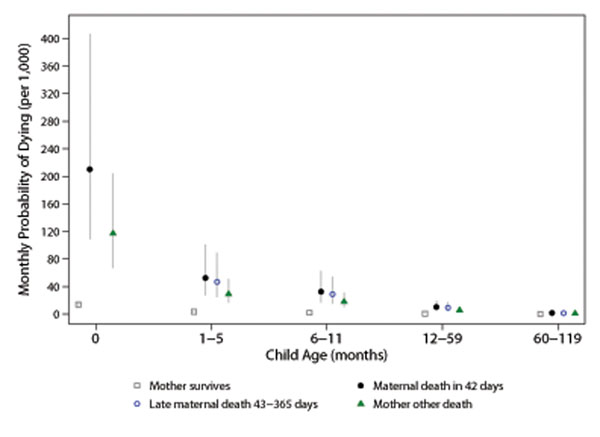
Monthly probability of child death by child age and mother’s survival status, Agincourt health and demographic surveillance system, South Africa, 1992 – 2013. Early maternal death defined as death during pregnancy, childbirth, or within 42 days of most recent childbirth or identified cause of death, late maternal death as death from 43 to 365 days after childbirth, and other death as any other death after 365 days of most recent childbirth. Adjusted for clustering of children with the same mothers, year, maternal age, and sex of child. Jittered points to reduce over plotting.

The monthly probabilities of dying associated with the relative risk ratios for mother’s survival status (Table 2, Model A) are shown in Figure 1. Children under one month of age whose mother died an early maternal death were at significantly greater risk of dying compared to children ages 1–5 months whose mother died an early maternal death (p=0.002). The risk of dying continued to decline with child age.

We next tested if household SES moderated the effect of mother’s survival status on a child’s risk of dying. Table S1 presents the same regression as Table 2 (Model A) but including an additional control for household SES [see Additional file 1]. While household SES was an important predictor of child mortality, it did not explain the effect of mother’s survival status.

### Cause of death

Model B in Table 2 includes the interaction of mother’s survival status with whether she died due to HIV/AIDS or TB or any other cause. Children who had a mother die of a HIV/AIDS or TB-related early maternal death were at 29 times the risk of dying (RRR 29.2 95% CI 11.7–73.1) relative to children whose mother survived. The risk of these children dying was significantly higher than those children whose mother died of a HIV/AIDS or TB-related non-maternal death (p=0.017). The next highest risk was for children whose mother died of a HIV/AIDS or TB-related late maternal death (RRR 14.3 95% CI 6.9–29.7).

Table 3 shows classifications of child deaths according to their mother’s survival status. Children whose mothers died a late maternal death more commonly died due to HIV/AIDS or TB (p = 0.010). Neonatal deaths were more common for children whose mothers died an early maternal death (p = 0.005).

**Table 3:** Child causes of death according to mother’s survival status, Agincourt health and demographic surveillance system, South Africa, 1992 – 2013. Data reported as number (%). Early maternal death defined as death during pregnancy, childbirth, or within 42 days of most recent childbirth or identified cause of death, and late maternal death as death from 43 to 365 days after childbirth. Child causes of death classified by InterVA-4 based on VA interviews.

	Survives (n= 1703)	Early maternal death (n= 14)	Late maternal death (n= 13)	Other death (n= 17)	p-value
HIV/AIDS or TB	398 (23)	1 (7)	7 (54)	7 (41)	0.010
Diarrhoeal diseases	155 (9)	0 (0)	2 (15)	1 (6)	0.565
Respiratory infections	338 (20)	3 (21)	2 (15)	0 (0)	0.166
Other infectious diseases	100 (6)	0 (0)	0 (0)	1 (6)	1.000
Nutritional disorders	27 (2)	0 (0)	0 (0)	0 (0)	1.000
Neonatal	233 (14)	6 (43)	0 (0)	0 (0)	0.005
External	62 (4)	0 (0)	0 (0)	0 (0)	1.000
Other	49 (3)	1 (7)	1 (8)	0 (0)	0.256
Unknown	341 (20)	3 (21)	1 (8)	8 (47)	0.041

## Discussion

Our results from a 22-year period in rural South Africa – with a population heavily burdened by HIV/AIDS – found that maternal death elevated children’s risk of dying and that the associated mortality risks were higher for very young children. A mother dying of an HIV/AIDS or TB-related maternal death further increased mortality risk, and their children were more likely to die due to HIV/AIDS or TB as well, relative to other causes.

The magnitudes of the adjusted association of children’s risk of dying following a maternal death are similar to those reported from other settings [34,35,4]. This analysis confirms other findings and further contributes to the evidence base of the consequences of maternal mortality for child survival in an HIV-endemic population. However, the levels of child mortality following a maternal death found in this study are lower than reported by some others [34,35]. Part of the explanation may be the cultural norms and traditions in this setting, by which the extended family provides support and care for orphaned children [36,37]. Further, there is a well-established social protection system in South Africa, with grants for children and older people that may enable families to support at-risk children [36,38].

Clinics within the study area began providing antiretroviral therapy (ART) in 2007 – by early 2009 1,500 patients were on ART provided by these public clinics [39]. There is no charge for ART, and these facilities are all within 10 km of each household. This ART rollout likely impacts both maternal and child survival [40,19] – our results suggest a decline in child mortality since 2011 that is consistent with the effect of ART, however further research is needed to examine this in detail.

The findings from this study – of increased risk of dying for children impacted by maternal death – highlight the need to address the causes of maternal mortality and protect surviving children. Sustained or strengthened investment in sexual and reproductive health, including adequate emergency obstetric care are needed, as well as providing access to contraception [41,42]. These strategies extend beyond maternal health, with improved maternal survival and newborn care enhancing the survival and well-being of young children and their families [43,44].

More robust governmental responses are particularly needed in rural, poor areas which tend to be underserved and where data are lacking, as well as in populations with high HIV/AIDS prevalence [42,11]. Providing access to information on contraception, prevention of mother-to-child transmission programs and early testing of infants, and access to antiretroviral therapy are all especially important factors in populations with high HIV/AIDS burden [45]. Given the prominence of maternal deaths due to AIDS or TB in this setting [46], management of HIV before pregnancy and in pregnant women is critical [12] – South African policy now places HIV-positive pregnant women on antiretrovirals irrespective of CD4 cell count [47]. The overriding risk to neonates and infants, both when their mother is very ill or recently died [8], highlights the importance of targeting these mother-infant pairs with community-based health workers. This is a central feature of the new policy on re-engineering primary health care in South Africa [48], with several examples of community-based interventions that have supported children affected by HIV/AIDS in sub-Saharan Africa [49].

We acknowledge limitations to the data and analytical approach used in this study. We focused on survival and ignored other aspects of parenting and the household that may affect other aspects of child development, such as successful schooling or adequate nutrition. Our findings use data from a defined region in South Africa – future studies are needed to generalize to other settings. Routine HIV testing is also not available for this study, limiting our ability to investigate the direct impact of HIV – instead we have used validated VA interviews [22] and InterVA to assign causes of death to children and mothers. In general the VA method does not identify highly specific causes of death well, and separating HIV from TB is particularly difficult using VA data [28,50]. We combine causes that InterVA-4 identifies as HIV and TB in order to address this fundamental limitation of the VA approach. Consequently we cannot identify separate HIV and TB effects. The site employs careful quality control measures and robust data checks to limit the possibility of missing vital events. This includes detailed questioning during each census update (including follow-up if a household member is now absent) and information on pregnancy status routinely collected and extensive training for fieldworkers to probe for pregnancy outcomes at the next census round.

## Conclusions

An important contribution of this study was to examine the relationship between maternal and child mortality in a population heavily burdened by HIV/AIDS. The intersecting epidemics of HIV infection and maternal health – especially in sub-Saharan Africa – have profound implications for both maternal and child mortality [9]. Our study provides policy-relevant evidence on children’s risk in the face of maternal death. These findings highlight the need to improve reproductive health services, particularly for HIV-positive women, as well as early identification and support for at-risk children.

## Competing interests

The authors declare that they have no competing interests.

## Author’s contributions

BH helped design the study, performed the statistical analysis, and drafted the manuscript. SJC helped conceive and design the study, contributed to draft revisions, and coordinated the research. KK and ST contributed to draft revisions. AEY is the PI on the overall qualitative-quantitative study on the four countries, and helped conceive the study and contributed to draft revisions. All authors read and approved the final manuscript.

## Funding

This project was conducted with support from The John and Katie Hansen Family Foundation. Thanks are also due to key funding partners of the MRC/Wits Rural Public Health and Health Transitions Research Unit who have enabled the ongoing Agincourt health and socio-demographic surveillance system: the Wellcome Trust grants 058893/Z/99/A, 069683/Z/02/Z, and 085477/Z/08/Z, UK; the Medical Research Council, University of the Witwatersrand, and Anglo-American Chairman’s Fund, South Africa; the Andrew W. Mellon Foundation, the William and Flora Hewlett Foundation, and the National Institutes of Health (NIH) grants K01 HD057246 from the Eunice Kennedy Shriver National Institute of Child Health and Human Development (NICHD) and R24 AG032112 from the National Institute on Aging (NIA), USA. The funders had no role in study design, data collection and analysis, decision to publish, or preparation of the manuscript.

## Supplementary Material

Additional file 1 Table S1. Relative risk regression of child death on child and mother characteristics and household SES, Agincourt health and demographic surveillance system, South Africa, 2001 – 2013 Click here for file

Additional file 2 Click here for file

## Declarations

This article has been published as part of *Reproductive Health* Volume 12 Supplement 1, 2015: True costs of maternal death. The full contents of the supplement are available online at http://www.reproductive-health-journal.com/supplements/12/S1. Publication charges for this supplement were funded by Family Care International and the FXB Center for Health and Human Rights.
