# Supplementary material for: The impacts of maternal mortality and cause of death on children’s risk of dying in rural South Africa: evidence from a population based surveillance study (1992-2013)
Source: Reprod Health. 2015 May 6;12(Suppl 1):S7. doi: 10.1186/1742-4755-12-S1-S7 (PMC4423728; doi:10.1186/1742-4755-12-S1-S7)
Supplement: Additional file 1 — Table S1. Relative risk regression of child death on child and mother characteristics and household SES, Agincourt health and demographic surveillance system, South Africa, 2001 – 2013 [file 1742-4755-12-S1-S7-S1.pdf]

**Table S1 Relative risk regression of child death on child and mother characteristics and household SES, Agincourt health and demographic surveillance system, South Africa, 2001 – 2013 (n=1,397,087 child months). Adjusted for clustering of children with the same mothers. Early maternal death defined as death during pregnancy, childbirth, or within 42 days of most recent childbirth or identified cause of death, and late maternal death as death from 43 to 365 days after childbirth. Household SES based on an asset survey.**

| | Relative risk ratio | 95% CI | p-value |
|---|---|---|---|
| Male | 1.112 | [0.905, 1.365] | 0.313 |
| Child age (months) | | | |
| 0 | 5.102 | [0.710, 36.671] | 0.105 |
| 1-5 | 7.530 | [5.094, 11.133] | <0.001 |
| 6-11 | 3.557 | [2.578, 4.908] | <0.001 |
| 12-59 | 1.000 | [1.000, 1.000] | . |
| 60-119 | 0.209 | [0.158, 0.276] | <0.001 |
| Mother's age (years) | | | |
| 15-19 | 1.000 | [1.000, 1.000] | . |
| 20-24 | 1.000 | [0.754, 1.326] | 0.998 |
| 25-29 | 1.020 | [0.755, 1.377] | 0.899 |
| 30-34 | 0.895 | [0.634, 1.265] | 0.531 |
| 35-39 | 1.046 | [0.715, 1.529] | 0.817 |
| ≥40 | 0.795 | [0.461, 1.372] | 0.410 |
| Year | | | |
| 2001 | 1.000 | [1.000, 1.000] | . |
| 2002 | 1.985 | [1.138, 3.463] | 0.016 |
| 2003 | 1.323 | [0.737, 2.375] | 0.348 |
| 2004 | 1.450 | [0.812, 2.590] | 0.209 |
| 2005 | 0.899 | [0.481, 1.677] | 0.737 |
| 2006 | 1.693 | [0.954, 3.006] | 0.072 |
| 2007 | 1.728 | [0.982, 3.042] | 0.058 |
| 2008 | 2.122 | [1.201, 3.750] | 0.010 |
| 2009 | 1.250 | [0.649, 2.406] | 0.504 |
| 2010 | 0.379 | [0.137, 1.045] | 0.061 |
| 2011 | 0.806 | [0.326, 1.993] | 0.640 |
| 2012 | 0.919 | [0.301, 2.806] | 0.882 |
| 2013 | 2.034 | [0.455, 9.104] | 0.353 |
| Asset quintiles | | | |
| Poorest | 1.000 | [1.000, 1.000] | . |
| Less poor | 0.613 | [0.454, 0.827] | 0.001 |
| Middle | 0.487 | [0.347, 0.684] | <0.001 |
| Richer | 0.627 | [0.465, 0.846] | 0.002 |
| Richest | 0.535 | [0.388, 0.736] | <0.001 |
| Mother's survival status | | | |
| Survives | 1.000 | [1.000, 1.000] | . |
| Early maternal death | 8.819 | [1.098, 70.864] | 0.041 |
| Late maternal death | 8.298 | [1.806, 38.122] | 0.007 |
| Other death | 6.178 | [2.862, 13.340] | <0.001 |
