# Supplementary material for: The impacts of maternal mortality and cause of death on children’s risk of dying in rural South Africa: evidence from a population based surveillance study (1992-2013)
Source: Reprod Health. 2015 May 6;12(Suppl 1):S7. doi: 10.1186/1742-4755-12-S1-S7 (PMC4423728; doi:10.1186/1742-4755-12-S1-S7)
Supplement: Additional file 2 [file 1742-4755-12-S1-S7-S2.pdf]

Referee's comments to the authors– this sheet WILL be seen by the author(s) and published with the article

Title	The impact of maternal mortality on children's risk of dying in rural South Africa: evidence from a population-based surveillance study (1992-2013)
Author(s)	Brian Houle, Samuel Clark, Kathleen Kahn, Stephen Tollman and Alicia Ely Yamin
Referee's name	Basia Zaba

When assessing the work, please consider the following points, where applicable:

1. **Is the question posed by the authors new and well defined?**
2. **Are the methods appropriate and well described, and are sufficient details provided to replicate the work?**
3. **Are the data sound and well controlled?**
4. **Does the manuscript adhere to the relevant standards for reporting and data deposition?**
5. **Are the discussion and conclusions well balanced and adequately supported by the data?**
6. **Do the title and abstract accurately convey what has been found?**
7. **Is the writing acceptable?**

Please make your report as constructive and detailed as possible in your comments so that authors have the opportunity to overcome any serious deficiencies that you find and please also divide your comments into the following categories:

- Major Compulsory Revisions (which the author must respond to before a decision on publication can be reached)
- Minor Essential Revisions (such as missing labels on figures, or the wrong use of a term, which the author can be trusted to correct)
- Discretionary Revisions (which are recommendations for improvement but which the author can choose to ignore)

Where possible please supply references to substantiate your comments.

When referring to the manuscript please provide specific page and paragraph citations where appropriate.

General comments: Although the question is not new, the evidence for the impact of maternal death on child mortality has not always been interpreted soundly, and it is useful to have a thorough investigation in a community with high HIV mortality, where the relationship may be different, although the lack of information on HIV status of living mothers is a drawback, which the authors acknowledge. The data collection methods are sound, and the analysis methods are appropriate and described clearly and in detail. Basic demographic data for the Agincourt study are available from the INDEPTH network iShare repository, but this is not mentioned in the paper. The standard of writing is excellent, discussion and conclusions are pertinent and well justified by the analysis results, and the title and abstract are informative.

Major compulsory revisions: none

Minor essential revisions: none

Discretionary revisions:

- Could mention data availability through iShare and through Agincourt's own sample data sharing scheme.
- A strength of this paper is the fact that the analysis allows for the impact of an imminent maternal death on child survival, i.e. the impact on child mortality of a mother's terminal illness, even if the child's death precedes that of the mother. This can be seen in figure 1, which illustrates, inter alia, the impact of a late

(continue on the next sheet)

Continued:

maternal death (43 days to 365 days following delivery) on mortality in children less than 1 month old. The fact that this type of impact is included could be specifically mentioned (e.g. in background section), since it is often neglected in more superficial analyses that only focus on what happens to children after the death of the mother. Linked to this, it might be useful to mention in the methods section how right censoring of mother's survival was dealt with: appropriately, maternal deaths that occurred 1 year or more after the child's birth have been grouped into a category "other", but it has not been made clear whether the "mother survives" category is restricted to mothers who could be observed as alive for at least 1 year after they gave birth, with shorter observed survival times censored by the end of the observation period.

- It might help to include a reference to a paper on HIV and maternal mortality: Zaba, Calvert, Marston et al. 2013. Effect of HIV infection on pregnancy-related mortality in sub-Saharan Africa: secondary analyses of pooled community-based data from the ALPHA network. Lancet, Vol 381 pp 1763-1771. This paper gives a direct estimate of HIV attributable pregnancy-related mortality in infected women, which could be used to gauge the population attributable effect in this community, given an estimate of HIV prevalence, which can be compared to the proportion of deaths diagnosed as HIV-related obtained from Verbal Autopsy. This paper also discusses the "healthy pregnant woman effect", which explains why the impact of HIV on maternal mortality is lower than the impact on the female population as a whole.

Referee's comments to the authors– this sheet WILL be seen by the author(s) and published with the article

Title	The impact of maternal mortality on children's risk of dying in rural South Africa: evidence from a population-based surveillance study (1992-2013)
Author(s)	Brian Houle, Samuel Clark, Kathleen Kahn, Stephen Tollman and Alicia Ely Yamin
Referee's name	Marie-Louise Newell

When assessing the work, please consider the following points, where applicable:

1. Is the question posed by the authors new and well defined?
2. Are the methods appropriate and well described, and are sufficient details provided to replicate the work?
3. Are the data sound and well controlled?
4. Does the manuscript adhere to the relevant standards for reporting and data deposition?
5. Are the discussion and conclusions well balanced and adequately supported by the data?
6. Do the title and abstract accurately convey what has been found?
7. Is the writing acceptable?

Please make your report as constructive and detailed as possible in your comments so that authors have the opportunity to overcome any serious deficiencies that you find and please also divide your comments into the following categories:

- Major Compulsory Revisions (which the author must respond to before a decision on publication can be reached)
- Minor Essential Revisions (such as missing labels on figures, or the wrong use of a term, which the author can be trusted to correct)
- Discretionary Revisions (which are recommendations for improvement but which the author can choose to ignore)

Where possible please supply references to substantiate your comments.

When referring to the manuscript please provide specific page and paragraph citations where appropriate.

General comments:
This paper addresses the association between death in the mother due to maternal causes and her child's risk of dying, with data from a large and ongoing demographic surveillance in rural South Aifrca. The paper is a follow-up of an earlier publication from the group, extending the analysis to cause of death and the age group of the children to 10 years. There are some parts of the methods which are not clear, in particular the issue of timing of death of the mother in relation to that of the child.

Major compulsory revisions:

The title should be amended to clarify where this paper differs from the earlier published paper – perhaps refer to cause of death and the age group of the children and the time period of data collection.

Abstract needs clarification regarding terms used and results provided. For example, is maternal death a death in a mother or a death due to maternal causes? What is early and what is late maternal death? What are the other causes of death in the mother? Was the RRR for an HIV death greater than for a non-HIV death?

Background: Clarify what is meant by Maternal Health in line 28, page 2. Is this as in pregnancy-related maternal health? Or health of mothers in general? The introduction needs to be clear about definitions of maternal death as there currently seems to be confusion between pregnancy-related death and death in mother, with the term 'maternal death' apparently relating to either.

Clarify in background or in methods whether deaths in mothers after the 'maternal/pregnancy-related' period are ignored?

Methods – page 4. InterVA is a weighted probability score of likely causes of death – discuss how this may or may not affect the findings of this paper

Results: page 5, line 29: the numbers of 71418 live births are not in Table 1? Clarify too the number of multiple births – there are more children than mothers.

Results: page 6, line 7: this sentence says that Male children had the same risk of dying as females, but the RR in Table 2 was 1.12, with a P-value of <0.0025, which suggests that boys were more likely to die than girls?

I am unclear about the timing of mother's death in relation to child death. See Results: page 6, line 28 (and methods) – please clarify that the risk of death for the child was only assessed after the mother had died? In particular I am confused about the increased risk of dying for children under one month of age whose mother died a late maternal death – in that case the child would have died before the mother, which is a difference scenario of a maternal death having an impact on child risk of dying?

Also clarify what happens with 'other (non-maternal) causes of death –are they completely ignored?

Page 7, line 16 – clarify when the HIV or TB child deaths occurred in children of mothers who died a late maternal death – and confirm the child death occurred after the maternal death – this then would be a maternal death linked to HIV with the child dying of HIV because of mother-to-child transmission?

Figure 1 – clarify here and in the methods when the mother other death would have occurred. Also, in the first set of monthly probabilities of dying, the child would have died before the mother died of a later maternal death – this seems strange (as already noted earlier).

In the discussion the authors may like to be clearer as to whether it is the timing of the maternal death (by definition these happen in early life and within the first year) or the cause that matters most. The fact that a maternal death occurs early when the risk for the infant will always be high and where alternative care may not be immediately available (especially not for breastfeeding) may be more important than the actual cause of death**.**

There could be more of a discussion of the trend in child deaths over the years (Table 2) – it is of interest that the more recent rates are not significantly different from those in the early part of the surveillance and indicates that with ART, the impact of HIV on mortality is diminishing.

Page 9, line 3 – what effect does the probability approach of InterVA have on the allocation of causes of death for this analysis?

Minor essential revisions:

Page 3, lines 14-22: AIDS is a consequence of HIV, the epidemic is an HIV epidemic.

Page 3, line 17 – HIV may be associated with global maternal mortality but whether it is a considerable contributor (implying strong causality) is a matter of debate.

Table 2: for the variable Child age is says (years) but that should be (months)

Page 6 – Maternal death – needs to refer to Table 2B rather than Table 2.

Page 7, line 11: please clarify where in the Table 2b, the p=0.017 should be.

The References need attention regarding formatting and detail. Often capitals are lower case, years are missing or page numbers.
